# Targeted Lung Premetastasis Niche: Mechanisms, Strategies, and Application

**DOI:** 10.1002/mco2.70248

**Published:** 2025-06-03

**Authors:** Chenghao Cao, Di Lu, Huigang Li, Shen Pan, Jianyong Zhuo, Zuyuan Lin, Chiyu He, Peiru Zhang, Songmin Ying, Xuyong Wei, Shusen Zheng, Zhe Yang, Xiao Xu

**Affiliations:** ^1^ Zhejiang University School of Medicine Hangzhou China; ^2^ School of Clinical Medicine Hangzhou Medical College Hangzhou China; ^3^ Department of Hepatobiliary and Pancreatic Surgery Hangzhou First People's Hospital Hangzhou China; ^4^ Department of Gastrointestinal Surgery The First Affiliated Hospital Zhejiang University School of Medicine Hangzhou China; ^5^ The Fourth School of Clinical Medicine Zhejiang Chinese Medical University Hangzhou China; ^6^ Department of Respiratory and Critical Care Medicine Second Affiliated Hospital of Zhejiang University School of Medicine Hangzhou China; ^7^ Department of Hepatobiliary and Pancreatic Surgery Key Laboratory of Artificial Organs and Computational Medicine in Zhejiang Province Shulan (Hangzhou) Hospital Shulan International Medical College Zhejiang Shuren University Hangzhou China; ^8^ Institute of Translational Medicine Zhejiang University School of Medicine Hangzhou China

**Keywords:** clinical implication, lung metastasis, premetastatic niche, therapeutic strategy, tumor microenvironment

## Abstract

Lung metastasis remains a leading cause of cancer mortality, driven not only by primary tumors but crucially by dynamic interactions within the premetastatic niche (PMN). Emerging evidence reveals that organ‐specific PMN formation precedes tumor cell arrival, creating an immunosuppressive microenvironment conducive to metastatic colonization. Primary tumor‐derived factors interact with lung resident cells, promoting the recruitment of bone marrow‐derived cells and creating a favorable microenvironment for the migrating tumor cells. Despite progress, systematic integration of PMN's multilayered regulatory mechanisms—from mechanism to clinical application remains lacking. The review summarizes recent studies investigating the effects of PMN in the entire process of lung metastasis. Our discussion revolves primarily around extracellular vesicles, extracellular matrix remodeling, proinflammation, immunosuppression, angiogenesis, and metabolic reprogramming. We emphasize the impact of various treatment strategies for primary tumors on the PMN in clinical practice, while also summarizing current diagnostic methods and exploring new therapeutic possibilities in the PMN. Finally, we propose a roadmap for future investigations into the lung PMN, highlighting prioritized research axes that bridge mechanistic discoveries with bench‐to‐bedside translation of PMN‐targeted interventions.

## Graphical abstract

Before the cancer cells arrive to the lung through blood vessels, these cancer cells secreted specific factors for two main purposes: first, to recruit bone marrow‐derived cells; and second, to regulate the activation of target cells. Additionally, the unique structure of the lungs provides a comfortable environment. Consequently, the formation of lung PMN is initiated, ultimately promoting organotropism.

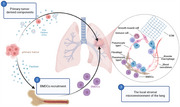

## 1 Introduction

The conceptual framework of cancer hallmarks, first proposed by Hanahan and Weinberg, identifies six fundamental biological capabilities driving tumor progression: sustained proliferative signaling, evasion of growth suppressors, resistance to cell death, replicative immortality, induction of angiogenesis, and activation of invasion and metastasis [1]. The lungs are a common site of metastatic dissemination, and it is noteworthy that most patients with cancer die because of their metastatic condition rather than the primary tumors [2, 3]. Clinically detectable lung metastasis is largely considered incurable, with only a few exceptions, owing to the acquired resistance of metastatic tumors to currently available therapies. This therapeutic impasse requires a deeper understanding of the spatiotemporal dynamics governing metastatic organotropism. As the broad relevance of these concepts grows, they are expected to increasingly influence the development of novel therapeutic strategies for the treatment of lung metastasis.

Technological advances in single‐cell RNA sequencing (scRNA‐seq) and digital spatial profiling have deconvoluted the tumor microenvironment (TME), revealing the complex crosstalk between neoplastic cells and lung stromal components [4, 5, 6, 7, 8]. Once considered as bystanders of tumorigenesis, these lung‐resident cells are currently recognized as key contributors to cancer pathogenesis [3]. Significantly, the influence of a developing tumor on the host extends beyond the confines of the local TME. Several premetastatic alterations have been identified in the lung [2]. Seminal work by Kaplan et al. [9] introduced the premetastatic niche (PMN) paradigm, a permissive microenvironment established in distant organs prior to tumor cell arrival. The PMN forms a microenvironment that supports metastasis, established within an organ without tumor cells. It primes remote locations for the eventual infiltration of disseminated tumor cells (DTCs). This has fundamentally altered the perception of metastasis. Recently, there has been a growing focus on the PMN hypothesis. However, systematic integration of these multiscale regulatory networks within lung PMN remains fragmented in the current literature.

This review synthesizes two decades of lung PMN research through a translational lens. We first delineate the evolutionary trajectory of PMN concepts from Paget's “seed and soil” hypothesis (1889) to modern omics‐era models. Subsequently, we provide an overview of multiple routes and mechanisms underlying lung PMN formation: tumor‐derived messengers: extracellular vesicles (EVs), extracellular matrix (ECM) remodeling, proinflammation, immunosuppression, angiogenesis, and metabolic reprogramming. Crucially, we evaluate ongoing clinical trials targeting lung PMN, providing evidence‐based assessment of innovative therapeutic prospects. The concluding section proposes future research priorities for lung PMN. Through this structured analysis, we aim to bridge mechanistic insights with clinical translation in metastatic lung cancer management.

## 2 “Seed and Soil” Hypothesis and Lung Organotropism

**FIGURE 1:**
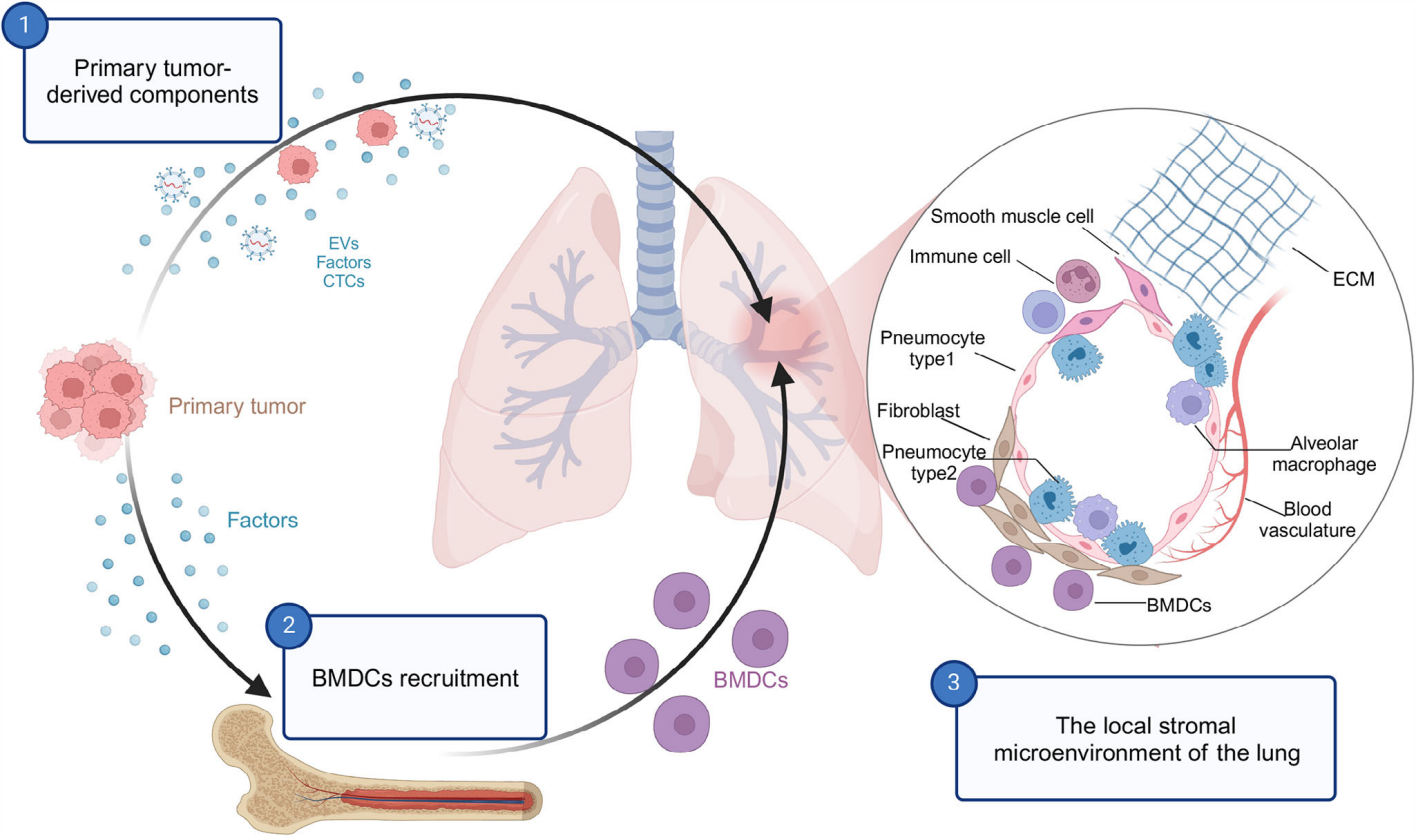
Formation of the lung PMN and requirements for metastatic organotropism. Before the cancer cells arrive in the lung through blood vessels, these cancer cells secrete specific factors for two main purposes: first, to recruit BMDCs; and second, to regulate the activation of target cells. Additionally, the unique structure of the lungs provides a comfortable environment for the metastasis of cancer cells. As a result of these actions, the formation of lung PMN is initiated, ultimately promoting organotropism.

In 1889, Stephen Paget suggested that metastasis relies on communication between certain cancer cells (referred to as the “seeds”) and specialized environments within organs (known as the “soil”). Paget's findings suggested that metastasis does not occur by chance (that time's prevailing viewpoint), but rather that certain tumor cells (the “seeds”) have a specific affinity for specific organ milieus (the “soil”) [10]. There has been substantial progress toward revealing the mechanisms governing tumor metastasis since the “seed‐and‐soil” hypothesis was proposed. Numerous studies have demonstrated the propensity for malignancies such as breast cancer, osteosarcoma, hepatocellular carcinoma (HCC), and melanoma to readily disseminate to the pulmonary organ. Recent studies have revealed that metastases in the lungs exhibit similar immune characteristics regardless of the original source of the primary tumor [11]. An increasing number of researchers are curious about why the lung organ (the “soil”) will be selected by tumor cells (the “seeds”). The lung provides a unique environment for metastasis, characterized by its airway and vasculature structures [12]. These structures intersect in the distal alveolars, enabling efficient gas exchange and the entry of circulating immune cells [13]. The PMN in the lung consists of smooth muscle cells, fibroblasts, and epithelial cells [14, 15]. Additionally, alveolar macrophages and bone marrow‐derived cells (BMDCs) are present in these niches [16, 17]. Various growth factors and cytokines from primary tumors contribute to the development of lung PMN [18] (Figure 1). Therefore, the organotropism is likely driven by the lung's unique microenvironment and the selectively enriched molecular and cellular components in different PMN.

## 3 Tumor‐Derived Messengers: EVs and Tumor‐Derived Soluble Factors

EVs represent notable factors that can actively participate in facilitating tumor‐stroma crosstalk, thereby facilitating the formation of lung PMN. Recent studies have revealed that EVs originating from tumor cells function as the bridge connecting primary tumors in their original location to secondary metastases, creating a conducive environment for the colonization and spread of tumor cells [19, 20]. Through kinetic analysis, scholars have found that the capture of EVs by lung resident stromal cells precedes the homing of metastatic tumor cells, consistent with the critical roles of EVs in the formation of PMN [21]. Fibroblasts and epithelial cells in the lungs are more likely to interact with the exosomes secreted by liver‐tropic exosomes [22]. These findings demonstrate that the capture of endogenously released EVs is organ specific.

Exosomes, sized between 30 and 150 nm, are intraluminal vesicles formed from the plasma membrane via the endosomal pathway and are secreted in a regulated manner. They contribute to the recruitment and reprogramming of cells through autocrine or paracrine signaling [23]. Exosomal RNAs, enriched with noncoding sequences, accumulate during endosome endocytosis. There are mainly three kinds of RNAs including miRNA [24, 25, 26, 27, 28], lncRNA [29, 30, 31], and snRNA [32] (Table 1). MicroRNAs (miRNAs), small noncoding RNAs that naturally evolve, negatively regulate gene expression by inhibiting or degrading target mRNA posttranscriptionally. For example, exosomal miR‐4508, miR‐25b‐3p, miR‐200b‐3p, and miR‐26‐5p contribute to the formation of the lung PMN [24, 26, 28, 33]. Interestingly, not all tumor cell‐derived EVs promote metastasis. Exosomes with low levels of let‐7s are necessary for the pluripotent factor Lin28B, which fosters lung metastasis by creating an immune‐suppressive PMN [34]. Lin28B facilitates neutrophil recruitment and the conversion to N2 neutrophils, which are crucial for immune suppression in the premetastatic lung through PD‐L2 upregulation and a disrupted cytokine environment [35]. EVs not only create a lung PMN for extrathoracic malignancies but also could indeed significantly enhance lung carcinoma cells’ intrapulmonary colonization, such as miR‐29a‐3p and exosomal miR‐3473b [25, 27].

**TABLE 1:** The role of extracellular vesicle's contents in lung PMN.

	Molecular	Function	Target	Tumor type with marked expression	Underlying mechanisms	References
RNA						
miRNA	miR‐4508	Promote	Lung fibroblasts	HCC	Promotes lung PMN formation by targeting the RFX1–IL17A–p38 MAPK–NF‐κB pathway	[24]
	miR‐26b‐5p	Promote	MDSC	ESCC	Enhances MDSCs expansion and activation by targeting phosphatase and tensin homolog in the lung PMN, thus selectively promoting pulmonary metastasis	[33]
	miR‐200b‐3p	Promote	MDSC	Breast carcinoma	Binds to PTEN, which may be involved in the regulation of AKT/NF‐κB/CCL2 cascades, recruiting the MDSCs in the lung and contributing to the establishment of the PMN	[28]
	miR‐25‐3p	Promote	ECs	CRC	Regulates the expression of VEGFR2, ZO‐1, occluding, and Claudin5 in ECs by targeting KLF2 and KLF4, consequently promoting vascular permeability and angiogenesis and enhancing CRC metastasis in lung PMN formation	[26]
	let‐7s	Inhibit	Neutrophil	Breast carcinoma	Decreases immune suppression by neutrophil recruitment and N2 neutrophils conversion in lung PMN	[35]
	miR‐29a‐3p	Inhibit	Lung fibroblasts	LLC	Significantly downregulates collagen I secretion by lung fibroblasts, contributing to the inhibition of lung metastasis	[27]
	miR‐3473b	Promote	Lung fibroblasts	LLC	Engulfs by lung fibroblasts and led to the NF‐κB signaling activation and, therefore, enhance the metastasis of lung tumor cells	[25]
	miR‐1270	Promote	ECs		Inhibits ZO‐1 expression in human ECs, which enhances their permeability, thus promoting the formation of lung PMN	[56]
LncRNA	MFI2‐AS1	Promote	miR‐107	NSCLC	Promotes cell proliferation, metastasis and invasion in a variety of malignancies, increases NFAT5 expression by sponging miR‐107, which in turn activates the PI3K/AKT pathway, promoting PMN formation	[30]
	PCAT‐1	Promote	miR‐182/miR217/fibroblast	Lung carcinoma	Increases expression of the immunosuppressive miR‐182/miR217 in lung tissues, thus promoting a PMN formation and a subsequent increase in lung metastatic burden; triggers fibroblast differentiation followed by CAF/myofibroblast secretion in TME triggering a CD133/SOX2‐related stem cell phenotype	[31]
snRNA		Promote	Lung epithelial cells	LLC/melanoma	Activates alveolar epithelial TLR3 to recruit neutrophils	[32]
Protein	Cav‐1	Promote	Lung epithelial cells/lung fibroblasts/lung macrophages	Breast carcinoma	Regulates the expression of PMN marker genes and inflammatory chemokines in lung epithelial cells, promotes the secretion of tenascin‐C (TnC) in lung fibroblasts to cause ECM deposition, and inhibits the PTEN/CCL2/VEGF‐A signaling pathway in lung macrophages to facilitate their M2‐type polarization and angiogenesis	[38, 40]
	ANXA6	Promote	ECs	Breast carcinoma	Promotes NF‐κB‐dependent EC activation, Ccl2 induction and Ly6C+CCR2+ monocyte expansion in the pulmonary PMN to facilitate the establishment of lung metastasis	[37]
	Rab22a‐NeoF1 fusion protein	Promote	Macrophages	Osteosarcoma	Recruits bone marrow‐derived macrophages	[39]
	PYK2	Promote	Macrophages	Osteosarcoma	Activates RhoA in its negative recipient osteosarcoma cells and induces signal transducer and activator of transcription 3 activation in its recipient macrophages to increase the M2 phenotype	[39]
	NID1	Promote	Fibroblasts	HCC	Enhances angiogenesis and pulmonary endothelial permeability to facilitate colonization of tumor cells and extrahepatic metastasis; activates fibroblasts, which secrete TNFR1, facilitate lung colonization of tumor cells, and augment HCC cell growth and motility	[42]
	Epiregulin	Promote	Vascular endothelial cell	SACC	Increases the expression of proangiogenic factors, such as VEGFA, bFGF, and IL‐8; enhances angiogenesis in the neighboring TME and increases vascular permeability in the premetastatic lung microenvironment	[43]
	NDPK	Promote	Vascular endothelial cell	Breast carcinoma	Promotes vascular EC migration and disrupts monolayer integrity in modulating the host microenvironment in favor of PMN formation	[44]

Abbreviations: carcinoma‐associated fibroblasts (CAFs); C‐C motif chemokine ligand 2 (CCL2); colorectal cancer (CRC); esophageal squamous cell carcinoma (ESCC); Insulin‐like growth factor 2 mRNA‐binding protein 1 (IGF2BP1); Lewis lung carcinoma (LCC); regulatory factor X1 (RFX1).

Long noncoding RNAs (lncRNAs) are a subclass of noncoding RNAs that span a length ranging from 200 nucleotides up to approximately 100 kilobases. They have been postulated to have a profound impact on diverse cellular processes, encompassing immune surveillance, regulation of the cell cycle, maintenance of stem cell pluripotency, and the intricate mechanisms underlying tumorigenesis [36]. Exosomal lncRNAs contributed to the formation of tumor‐associated PMNs and offered new insights into the molecular mechanisms underlying the cancer metastasis microenvironment [29]. For example, exosomal lncRNA MFI2 antisense RNA 1 (MFI2‐AS1) and lncRNA prostate cancer‐associated transcript 1 (PCAT‐1) promote PMN formation and a subsequent increase in lung metastatic burden [30, 31]. Exosomal RNAs derived from the primary tumor, containing a high concentration of snRNA (small nuclear RNAs), trigger the activation of Toll‐like receptor 3 (TLR3) in lung epithelial cells. As a result, this activation leads to the secretion of chemokines in the lung, thereby supporting the recruitment of neutrophils [32].

The production of exosomes is linked to the endocytic pathway, and it supports their endosomal origin. Consequently, the primary category of cargo proteins comprises membrane transport and fusion proteins such as Caveolin‐1 (Cav‐1), annexin A6 (ANXA6), Rab22a‐NeoF1, and proline‐rich tyrosine kinase 2 (PYK2) [37, 38, 39, 40] (Table 1). Tetraspanins, a class of transmembrane proteins found in exosomes, form a key component of the exosomal structure. The tetraspanin network acts as a molecular scaffold, facilitating signal transduction by linking various proteins and is involved in processes such as cell motility, adhesion, morphogenesis, and membrane fusion. The tetraspanin–integrin complex plays a role in directing cancer‐derived exosomes to the PMN [41]. In metastatic HCC, EV‐derived nidogen 1 (NID1) promotes PMN formation in the lung by enhancing angiogenesis and increasing endothelial permeability, aiding tumor colonization and extrahepatic metastasis. EV‐NID1 also activates fibroblasts, leading to tumor necrosis factor receptor 1 (TNFR1) secretion, which supports lung colonization and HCC cell growth [42]. A major component of tumor exosome‐regulated organotropic metastasis is angiogenesis and epithelial–mesenchymal transition (EMT). Upregulation of epiregulin in salivary adenoid cystic carcinoma (SACC) cells induced EMT by regulating GLI1/E‐cadherin [43]. EV‐associated nucleoside diphosphate kinase A and B (NDPK) and ANXA6 promoted vascular endothelial cell (EC) migration in the pulmonary PMN [37, 44]. EGFR and some of the cognate ligands extensively traffic in EVs. EV‐mediated EGFR signaling is important to EGFR‐driven cancers [45, 46].

Apart from the contents of EVs, there exist several factors that can facilitate their release. Mutant Tumor protein p53 (TP53) plays a crucial role in regulating the secretion, size, and particularly the RNA and protein cargo of tumor‐derived EVs. This process establishes a supportive microenvironment at the primary tumor site and prepares niches in distant organs for subsequent metastatic colonization [47, 48]. The generation of antitumor EVs relied on the acetylation of p53, facilitated by the BAG6/CBP/p300–acetylase complex. The BAG6/CBP/p300–p53 pathway serves as a crucial mechanism for directing the loading of cargo into EVs, making it a promising and innovative target for therapeutic intervention in the microenvironment [49]. Modifications induced by the Snail transcription factor on colorectal cancer cells, leading to a more invasive phenotype, also result in the alteration of the miRNA content within released EVs and influence the modifications of the PMN by promoting lung inflammation [50].

Likewise, by hampering the uptake of EVs, it is possible to induce alterations in the lung PMN. The antihypertensive drug reserpine suppresses EV uptake and disrupts EV‐driven PMN formation and melanoma lung metastasis [51]. Melanoma EVs downregulate type I interferon (IFN) receptors and IFN‐inducible cholesterol 25‐hydroxylase, reducing 25‐hydroxycholesterol production and inhibiting EV uptake. Activation of p38α also inactivates type I IFN signaling and increases fibroblast activation protein expression, which remodels the ECM and promotes chemokine production, aiding neutrophil infiltration into the lungs [52]. These neutrophils enhance PMN formation, facilitating tumor cell extravasation and proliferation through the upregulation of prometastatic proteins such as Bv8, MMP9, S100A8, and S100A9 [52, 53]. The use of p38 inhibitors shows potential in the adjuvant treatment of metastatic cancers.

Exosomes derived from melanoma cells with low metastatic potential exhibit a strong capability to suppress lung metastasis. Exosomes from melanoma cells with low metastatic potential effectively suppress lung metastasis by triggering an innate immune response. They expand low Ly6C patrolling monocytes (PMo) in the bone marrow, which clear cancer cells at the PMN by recruiting NK cells to remove tumor material from the lung vasculature. This process relies on the Nr4a1 transcription factor and the presence of pigment epithelium‐derived factor on the exosome surface [54]. PMo accumulate in the lung microvasculature, helping to reduce lung metastasis [55]. These findings may provide new mechanistic insights into how targeting EVs could be a potential strategy for curing metastatic progression in the lung.

The formation of the PMN also involves tumor‐derived soluble factors (TDSFs), including cytokines, growth factors and proteases [57] (Table 1). IL‐6, secreted by primary tumor cells and associated stromal cells, promotes PMN formation by activating STAT3 in tumor cells and tumor‐infiltrating myeloid cells [58, 59, 60, 61, 62, 63, 64, 65, 66, 67]. CXCR4, activated by its ligand CXCL12, plays a critical dual role in shaping the lung PMN. The CXCL12–CXCR4 axis drives the recruitment of CXCR4‐expressing tumor cells to CXCL12‐rich organs such as the lungs, facilitating metastatic seeding through chemotactic migration. Furthermore, TDSFs induce stromal cells within the PMN to produce CXCL12, establishing a chemotactic gradient that recruits immunosuppressive leukocytes—including neutrophils, MDSCs, and macrophages—to create a pro‐TME characterized by immune tolerance and stromal remodeling [68]. Additionally, tumor‐derived CCL2 recruits MDSCs and CD4+ cells to premetastatic organs [28, 69]. Studies have shown that vascular endothelial growth factor α (VEGF‐α) induces the expression of proinflammatory proteins S100A8 and S100A9, promoting the infiltration of Mac1+ myeloid cells into the pulmonary PMN [18, 70]. Further, lung fibroblast activation, driven by exosomal TGF‐β1 and IGF2BP1, is a critical event in PMN formation [63, 71–75]. Lysyl oxidase homolog 2 (LOXL‐2) remodels stromal components in target metastatic organs, increasing tissue stiffness and recruiting BMDCs [76, 77, 78]. Recent studies reveal that tumor‐secreted cathepsin C (CTSC) facilitates pulmonary tumor colonization by recruiting neutrophils and inducing neutrophil extracellular trap (NET) formation [79]. TDSFs hold significant promise as diagnostic biomarkers for early PMN detection and therapeutic targets to disrupt metastasis by blocking stromal‐immune crosstalk, angiogenesis, and neutrophil‐mediated niche formation (Table 2).

**TABLE 2:** The role of tumor‐derived secreted factors in lung PMN.

	Molecular	Function	Target	Tumor type with marked expression	Underlying mechanisms	References
Cytokine	IL‐6	Promote	Tumor cells/tumor‐infiltrating myeloid cells	Breast carcinoma	Promoting PMN formation by activating STAT3 in tumor cells and tumor‐infiltrating myeloid cells	[59, 61, 65, 66]
	CXCL12	Promote	Immunosuppressive leukocytes	Melanoma	Creating a chemotactic gradient that recruits neutrophils and myeloid cells and establishes a pro‐TME	[68]
	CCL2	Promote	MDSCs	Breast carcinoma	Increases CD4+ T cell recruitment to the PMN of the lung and this correlated with enhanced seeding and growth of tumor cells	[28, 69]
	TNF‐α	Promote	Macrophages	Lung carcinoma	Stimulates LECs activity through a VEGFR3‐independent mechanism but also induces inflammatory macrophages to produce high levels of VEGF‐C to coordinately activate VEGFR3, while VEGFR3‐induced LEC tips are a prerequisite for lymphatic vessel growth in vivo	[71, 73]
Growth factor	VEGF	Promote	BMDC	Lung carcinoma	Attracts VEGFR1+ bone marrow‐derived HPCs to the PMN prior to the arrival of tumor cells	[18, 70]
	TGF‐β1	Promote	Lung fibroblast	Osteosarcoma	Elicits an upsurge in invasive competence in human lung fibroblasts and increases α‐smooth muscle actin expression and fibronectin production	[75]
	IGF2BP1	Promote	CD45+ cells	Melanoma	Promotes fibronectin deposition and accumulation of CD45+ cells in the lungs compared with control EVs, thus creating the PMN formation potential of EVs	[74]
Proteases	LOXL2	Promote	BMDCs	HCC	Promotes fibronectin production, MMP9 and CXCL12 expression, and BMDCs recruitment to assist PMN formation	[76, 77, 78]
	CTSC	Promote	Neutrophils	Breast carcinoma	Activates neutrophil membrane‐bound PR3 to facilitate IL‐1β processing and NF‐ κB activation, thus upregulating IL‐6 and CCL3 for neutrophil recruitment	[79]

Abbreviations: lymphatic endothelial cells (LECs); hematopoietic progenitor cells (HPCs); proteinase 3 (PR3).

## 4 The Characteristic of Lung PMN

During the formation of the PMN in the lung, both the influence of primary tumors and the organ‐specific characteristics of the pulmonary PMN critically shape metastatic progression. As proposed by Cao et al., the PMN exhibits six hallmark features [80, 81]. However, compared with liver and bone—common metastatic target organs—the pulmonary PMN demonstrates unique organ‐adaptive properties [82]. Based on a synthesis of current literature, we highlight that the pulmonary PMN is collaboratively shaped by multidimensional dynamics, including ECM remodeling, proinflammatory and immunosuppression, angiogenesis/lymphangiogenesis, and metabolic reprogramming, which collectively establish a permissive “soil” for metastatic colonization. These organ‐specific adaptations enable tumor cells to breach the blood–air barrier and achieve lung‐tropic colonization through multifaceted molecular and biophysical support.

### 4.1 Remodeling the ECM

The ECM is a complex network of extracellular‐secreted macromolecules. It consists of collagen, proteoglycans, and glycosaminoglycans, whose main functions deal with structural scaffolding and biochemical support of cells and tissues [83]. In a state of malignancy, there is an intensified upregulation of multiple proteins that comprise the intricate framework of the ECM, resulting in the recruitment of secondary cells and remodeling to prepare for metastasis [84]. Cancer cells intricately choreograph a milieu conducive to tumor growth by enlisting and reprogramming nonmalignant host cells, as well as reshaping the vasculature and ECM. The progression of lung metastasis is intricately influenced by various soluble and insoluble factors present within the stromal environment. Soluble components such as chemokines, cytokines, and growth factors actively participate in this process, while insoluble constituents predominantly encompass the ECM. The ECM can serve as a structural framework for the retention of cells that comprise the PMN.

Higher levels of matrix stiffness have an impact on the biological characteristics of tumor cells, leading to the promotion of lung metastasis in multiple types of tumors [85]. An illustration of this phenomenon is the cell migration‐inducing hyaluronan‐binding protein, which can enhance the formation of PMN by increasing the stiffness of the lung matrix [86]. ECM remodeling following surgery renders the lungs more susceptible to metastasis. The release of ATP or UTP from hypoxia‐treated cancer cells triggers the expression of HIF‐1α and the secretion of LOX through the activation of the P2Y2 receptor. This process contributes to the induction of collagen crosslinking [87]. Crosslinked fibrillary collagen (such as crosslinked collagen IV) facilitates the adherence of CD11b+ cells, which in turn produce MMP2. This enzyme cleaves collagen and enhances the invasion and recruitment of both BMDCs and metastasizing tumor cells [88, 89, 90]. Under higher stiffness stimulation, LOX Like Protein 2 derived from HCC cells not only upregulates MMP9 and chemokine CXCL12 expression but also increases fibronectin production in lung fibroblasts. This is achieved through the activation of the PI3K–AKT pathway and the Integrin β1/α5/JNK/c‐JUN signaling pathway, thereby supporting the formation of lung PMN [77, 78]. Cancer cells have the ability to spread and establish themselves in distant organs, where they may remain inactive for extended periods of time before giving rise to clinically observable metastases. During this dormant phase, cancer cells create a specialized ECM environment rich in type III collagen. It has been determined that the presence of tumor‐derived type III collagen is crucial for maintaining tumor dormancy in patients, as its disturbance reactivates tumor cell growth via the DDR1‐mediated STAT1 signaling pathway [91, 92]. The PMN establishment of BMDCs creates favorable conditions for forthcoming metastasis. Versican, an ECM proteoglycan, is expressed by myeloid cells in metastatic pulmonary sites. Through reducing phospho‐Smad2 levels, versican promotes the mesenchymal to epithelial transition (MET) of metastatic tumor cells. Consequently, cell proliferation rises, and the emergence of metastases accelerates [93]. Fibroblasts play a central role in modifying the architecture and stiffness of ECM [94, 95]. Thus, antifibrotic strategies may be useful in suppressing lung metastasis [96].

### 4.2 Proinflammation

Inflammation is distinguished by the excretion of a multitude of proinflammatory cytokines and the infiltration of inflammatory cells into nearby tissues, constituting a pivotal feature of the PMN. A microenvironment with proinflammatory influences sparks a cascade of molecular components and signaling pathways, thereby inciting the permeability of blood vessels, augmenting tumor cell intrusion into the stromal tissue, reawakening dormant circulating tumor cells (CTCs), and fostering the manifestation of malignancy in tumor cells [84]. Currently, both primary tumor tissues and metastatic foci are thought to be accompanied by inflammation, a process that involves both leukocyte mobilization within the body [1]. In the absence of tumor cells, premetastatic lungs display a state of inflammation, attributed to the disruption of homeostatic innate immune responses.

Chemoattractants like S100A8 and S100A9 are calcium‐binding proteins induced by distant primary tumors and immune cells in the PMN, serving as fertile ground for metastasis [97, 98]. These proteins are implicated in modulating the TME to an immunosuppressive state and are associated with tumor metastasis [18, 52, 53]. Serum amyloid A (SAA) 3, induced in lung PMN by S100A8 and S100A9, plays a role in myeloid cell accumulation and acts as a positive feedback regulator for chemoattractant secretion. IL‐1β derived from alveolar macrophages significantly boosts SAA3 expression, stimulating NF‐κB signaling in a TLR4‐dependent manner in lung ECs and macrophages [99], thereby increasing MMP9 levels in an autocrine manner and creating a conducive lung PMN environment, ultimately promoting pulmonary metastasis of HCC [100]. Moreover, S100A9‐targeted cowpea mosaic virus CPMV homes to the lungs, leading to the recruitment of innate immune cells, to prevent metastasis outgrowth, and potentially serves as a therapeutic option for established metastatic disease [101].

Undoubtedly, neutrophils play a significant role as acute inflammation effectors. Multiple lines of evidence suggest their involvement in chronic inflammatory conditions and adaptive immune responses as well [102]. An increasing number of studies indicate that neutrophils play a significant role in cancer progression. Neutrophil polarization to protumorigenic phenotype (N2) is driven by the TME [103]. An enhanced infiltration of N2‐type neutrophils and classical monocytes linked to chronic inflammation was discovered through single‐cell transcriptomic profiling of all lung cells. Particularly noteworthy is the similarity in N2‐type phenotypes and heightened expression of inflammatory and angiogenic factors in lung neutrophils [7]. Long‐term nicotine exposure significantly contributes to the development of PMN within the lungs by recruiting protumor N2‐neutrophils. This PMN stimulates the secretion of STAT3‐activated lipocalin 2 (LCN2), a secretory glycoprotein, from the N2‐neutrophils, thus triggering MET of tumor cells, consequently facilitating their colonization and metastatic expansion. These findings shed light on the potential therapeutic application of salidroside to enhance the antitumor efficacy of neutrophils in patients with breast cancer [104].

Neutrophils affect cancer through multiple mechanisms, and evidence for a role of NETs is also emerging [91, 105]. The formation of NETs, which are essential for triggering the reactivation of dormant cancer cells, was observed as a result of prolonged lung inflammation caused by exposure to tobacco smoke or the nasal instillation of lipopolysaccharide. Sequentially, NET‐associated proteases, including neutrophil elastase and MMP9, cleaved laminin [106]. This proteolytically modified laminin then stimulated the proliferation of dormant cancer cells by activating integrin α3β1 signaling [107]. The production of CXCL2, stimulated by chronic stress, facilitates the recruitment of neutrophils to the lungs. Additionally, it induces pulmonary epithelial cells to generate acetylcholine, which enhances the NETosis of neutrophils, thereby restructuring the lung PMN [108]. Lung mesenchymal stromal cells, in a STAT6‐dependent manner, promote the induction and maintenance of complement 3 (C3) through Th2 cytokines. This process further facilitates the recruitment of neutrophils and the formation of NETs. Ultimately, these NETs play a crucial role in promoting cancer cell metastasis to the lungs during the premetastatic stage [109]. Lung oxalate accumulation induced NETs formation by activating NADPH oxidase, which facilitated the formation of PMN [110]. Therefore, pursuing these avenues for targeting NETs could prove to be a promising therapeutic approach for lung metastasis.

### 4.3 Immunosuppression

One of the most intensely investigated premetastatic changes is via the induction of immunosuppression, favoring immune escape of DTCs [111]. In recent years, a substantial number of studies reported that a suppressive immune environment is forged before the colonization of CTCs, promoting tumor metastasis [112]. Some regulatory or immunosuppressive cells, such as tumor‐associated macrophages (TAMs), MDSCs, and regulatory T cells (Tregs) within the PMN, potentially suppress antitumor immune responses.

TAMs represent a major class of immunosuppressive cells that can be classified into two distinct phenotypes (M1 and M2) [113, 114, 115]. The M1 phenotype suppresses tumor growth, while the M2 phenotype has a supportive role in driving tumor progression [116]. Through modulation of the STAT6 signaling pathway, this approach effectively triggers TAM recruitment, impedes M2‐like macrophage polarization, and subsequently suppresses pulmonary metastasis [117, 118, 119, 120]. A single‐cell analysis encompassing various metastatic stages and regions demonstrated that inflammatory neutrophils and monocytes infiltrate premetastatic lungs, succeeded by the accumulation of suppressive macrophages upon the emergence of metastases. Spatial profiling indicated the unique enrichment of triggering receptor expressed on myeloid cells‐2 regulatory macrophages at the invasive margin of metastases. These regulatory macrophages play a role in establishing an immune‐suppressive niche, shielding tumor cells from immune surveillance [8].

Further, other BMDCs mobilized by tumors, such as neutrophils and monocytes, are educated to form an immunosuppressive polymorphonuclear population in the lungs [121]. Their expansion is driven by cytokines such as CXCL1, CXCL2, VEGF, and IL‐1β, mediated through transcriptional regulators like NF‐κB, while activation involves signaling pathways including TLR4/NF‐κB and COX‐2/PGE2, and endoplasmic reticulum stress [4, 122–125]. BMDCs play a central role in cancer metastasis by orchestrating the immunosuppressive PMN through ECM remodeling, such as the production of MMP9 and S100A8/A9, which facilitate tumor cell extravasation and colonization [126, 127]. Acting as a potential diagnostic biomarker for PMN formation, BMDCs can be detected using targeted imaging probes (e.g., Gr‐1‐binding nanoprobes) in high‐metastasis contexts, enabling early PMN identification and disease prognosis [128]. Therapeutic strategies targeting BMDCs focus on inhibiting their expansion and recruitment (e.g., blocking CCL2/CCR2) or suppressing their immunosuppressive functions via COX‐2 inhibitors, with enhanced efficacy observed when combined with immune checkpoint inhibitors (e.g., anti‐PD‐1/PD‐L1) [4, 124]. These approaches offer a transformative paradigm for intercepting metastasis by precision targeting of BMDC‐driven PMN dynamics. Current strategies, including BMDC inhibition, differentiation, functional blockade, and depletion, show promise in preclinical and early clinical studies, with future directions emphasizing the standardization of biomarker‐driven therapies, integration of multiomics approaches, and optimization of combinatorial regimens (e.g., ICBs + BMDC‐targeted agents) to translate metastatic interception into clinical reality.

Tregs, an immunosuppressive cell type widely present in tumors, play a vital part in PNM formation [129]. Elevated levels of CCL1 secreted by lung fibroblasts prompted Treg differentiation through the activation of its dedicated receptor CCR8, ultimately playing a role in establishing an immunologically tolerant PMN [130]. Breast cancer cell‐secreted GM‐CSF activates the STAT5 signaling pathway to stabilize aryl hydrocarbon receptor in lung macrophages by blocking its ubiquitination, which subsequently drives PD‐L1 upregulation via direct binding to the PD‐L1 promoter, thereby promoting Treg differentiation—a pivotal mechanism for establishing the immunosuppressive lung PMN critical to breast cancer metastasis [131]. The combined inhibition of Treg proliferation, activation of effector T cells, and suppression of tumor‐promoting factor secretion synergistically impede metastatic progression [132]. In summary, the PMN, characterized by inflammation and immunosuppression, may mediate interconnected mechanisms to facilitate successful tumor metastasis.

### 4.4 Angiogenesis and Lymphangiogenesis

Angiogenesis, an important feature of PMN, is essential for tumor growth, invasion, and metastasis. Once a tumor surpasses a size of approximately 1–2 mm, it becomes imperative for it to forge its vascular conduit, facilitating the supply of vital oxygen and nutrients. The intricate web of vasculature assumes a pivotal role in furnishing tumors within the TME with the essential sustenance they require [133]. Lung tissue contains numerous vascular endothelial and epithelial cells that form the air‐blood barrier [134]. The establishment of a dense capillary network and enhanced vascular permeability are necessary for the extravasation and survival of metastatic cancer cells.

VEGF from the primary tumor stimulates PGE2 production in mouse pulmonary microvascular ECs (MPVECs), promoting tumor cell adhesion and triggering an inflammatory response that alters the lung microenvironment in the premetastatic phase. Celecoxib, a COX‐2 inhibitor, significantly reduces tumor cell adhesion to MPVECs, as well as cancer metastasis and the associated inflammation [135]. Lymphatic vessel formation in the lung PMN is orchestrated through synergistic molecular and cellular mechanisms involving TDSFs, immune modulation, and EV‐mediated reprogramming. LECs express specific markers such as VEGFR‐3 and prospero homeobox 1 (PROX1), which regulate lymphatic vessel sprouting [9, 65, 136]. Tumor‐derived VEGF‐C/VEGFR‐3 signaling drives lymphangiogenesis by activating LECs, while VEGFR1+ hematopoietic bone marrow progenitors colonize premetastatic sites to secrete prolymphangiogenic factors, priming the niche for metastasis [136]. Single‐cell transcriptomic studies reveal that tumor‐associated neutrophils (TANs) infiltrating the PMN enhance LEC proliferation and lymphatic sprouting via ERK/JNK‐mediated secretion of MMP9 and VEGFA [7]. Additionally, tumor EVs deliver molecular cargo (e.g., NGFR in melanoma, SUMOylated hnRNPA1 in pancreatic cancer, and circTLCD4‐RWDD3 in lung cancer) to LECs, upregulating PROX1 and inducing lymphatic network expansion [137]. Immune suppression by VEGFR1+ progenitors and tumor cells further establishes an immune‐tolerant microenvironment conducive to lymphatic metastasis. Therapeutic targeting of these pathways, such as apatinib (a VEGFR inhibitor), demonstrates potential to disrupt PMN formation, as evidenced in clinical trials for metastatic triple‐negative breast cancer [138]. Inhibition of cytochrome P450 4A reduces lung PMN formation by decreasing VEGFR1+ myeloid cell recruitment and prometastatic protein expression [139].

Interestingly, some studies have revealed that cancer cells resistant to chemotherapy or tyrosine kinase inhibitors can induce lung metastasis through the remodeling of vascular structures. Paradoxically, the increase in tumor metastasis, despite sunitinib being designed to directly target ECs by inhibiting multiple receptors such as VEGFRs, may be attributed to sunitinib effectively fulfilling its expected role. The senescence of ECs resulted in a significant increase in the secretion of inflammatory chemokines and the expression of vascular cell adhesion molecule‐1, thereby promoting chemotaxis of tumor cells and interactions between tumor cells and ECs. Meanwhile, the senescence of ECs led to the disruption of EC junctions, facilitating the transmigration of tumor cells across the endothelial barrier. Sunitinib induced a senescence‐like EC phenotype. Additionally, the senescent ECs induced by sunitinib attracted cancer‐associated myeloid cells, contributing to the formation of a PMN. These alterations at both the molecular and tissue levels ultimately led to an elevated occurrence of distant metastasis [140]. Integrated multiomics approaches, including radiomics and transcriptomics, corroborate the role of lymphatic‐driven molecular traits in metastasis, highlighting opportunities for precision intervention.

### 4.5 Metabolic Reprogramming

Metabolic reprogramming is shown to be involved in PMN to promote tumor metastasis. Lipid metabolic reprogramming is a developing characteristic of cancer [141, 142, 143]. Tumor cells undergo metabolic shifts to sustain uncontrolled proliferation and survival in oxygen‐ and nutrient‐deprived environments, exploiting diverse mechanisms to acquire and enhance lipid oxidation. Moreover, within the TME, stromal and immune cells also experience a shift in lipid metabolism, thus further influencing tumor functional phenotypes and immune responses [144]. Obesity is characterized by chronic systemic inflammation and enhances lung metastasis and mortality [145]. Lung is a lipid‐rich environment and high‐fat diet (HFD) promotes lung PMN formation and metastasis. Glycyrrhizic acid reduced HFD‐enhanced MDSCs recruitment and prometastatic protein S100A8/A9 expression through decreasing the proportion of M1‐like macrophages and their secretion of CCL2 and TNF‐α in the colons via inactivation of the LPS/HMGB1/NF‐κB signaling pathway [146]. PMN formation specifically increases the availability of palmitate in the lungs, while HFD elevates palmitate levels in both the lung and liver [147]. Environments rich in palmitate within lung PMNs promote the growth of metastases by boosting p65 acetylation, thereby activating prometastatic NF‐κB signaling [148]. The process of cholesterol synthesis plays a crucial role in promoting the development of lung metastasis [149]. It was possible to prevent the occurrence of lung metastasis by reducing cholesterol levels with atorvastatin [150]. While neutrophils from bone marrow or blood exhibit minimal immunosuppression, those infiltrating the lungs strongly suppress T and NK cells. This enhanced immunosuppression is driven by lung‐resident mesenchymal cells (MCs), which inhibit adipose triglyceride lipase (ATGL) activity in neutrophils via PGE2‐dependent and independent mechanisms. At the premetastatic stage, lung MCs accumulate neutral lipids, with fibroblasts producing PGE2, leading to dysfunctional dendritic cells (DCs) and suppressive monocytes. This process is partially regulated by IL‐1β‐induced hypoxia‐inducible lipid droplet‐associated, which reduces ATGL activity in MCs. Lipids stored in lung neutrophils are transferred to metastatic tumor cells through a macropinocytosis‐lysosome pathway, enhancing tumor cell survival and proliferation [4, 145, 151–154]. Oxysterols regulate lipid metabolism by interacting with nuclear liver X receptors (LXR) α and LXR β. In tumors, they create a protumor environment by suppressing antitumor immunity and attracting neutrophils that promote angiogenesis and immunosuppression. Additionally, oxysterols enhance metastasis by altering the lung PMN, facilitating the recruitment of neutrophils that support tumor growth [155].

The uptake of microparticles derived from tumors by macrophages triggers a swift metabolic and phenotypic transition, marked by heightened mitochondrial mass and function, elevated oxidative phosphorylation, and heightened expression of adhesion molecules. This ultimately leads to decreased motility in the early metastatic lung. This reprogramming is driven by the mTORC1 pathway, but not the mTORC2, pathway and is specifically triggered by the uptake of tumor microparticles [156]. Tumor‐derived EVs enriched with glycolysis‐related proteins (e.g., HK2, LDHA) induce glycolytic activation in alveolar type 2 cells, resulting in lactate accumulation and a pronounced pH drop within the lung PMN [157]. This acidosis, detectable via pH‐sensitive probes like pH Low Insertion Peptide (pHLIP), facilitates metastatic colonization, while pharmacological inhibition of glycolysis (e.g., 2‐DG) reverses acidification and suppresses metastasis. Additionally, pHLIP‐conjugated dexamethasone disrupts proinflammatory signaling in acidic PMN, underscoring the therapeutic potential of targeting glycolytic activity and pH imbalances. Concurrently, aspartate enrichment in lung interstitial fluid activates the NMDA receptor–CREB–DOHH–eIF5A axis in DTCs driving TGFβ‐dependent collagen synthesis through noncanonical translation mechanisms to enhance metastatic aggressiveness [158]. Complementing these findings, tumor‐derived exosomes polarize macrophages toward an immunosuppressive phenotype via TLR2/NF‐κB signaling, enforcing glycolytic dominance (increased glucose uptake and lactate production) while suppressing oxidative phosphorylation. Lactate stabilizes NF‐κB activity, upregulating PD‐L1 expression on macrophages and Tregs to establish immune tolerance [159]. Collectively, these studies reveal that glycolysis‐driven acidosis, aspartate‐mediated stromal remodeling, and exosome‐induced metabolic‐immune crosstalk synergize to create a metastasis‐permissive PMN [160]. Therapeutic strategies targeting glycolytic enzymes (e.g., LDHA), pH‐guided drug delivery (e.g., pHLIP conjugates), or exosome signaling pathways may disrupt PMN formation, offering innovative approaches to combat metastatic recurrence.

## 5 Clinical Implications

The organ‐specific characteristics of the lung PMN present both unique opportunities and challenges for clinical intervention. Building on the dynamic remodeling patterns of lung PMN following primary tumor therapy (surgical resection or systemic treatment), optimal clinical management necessitates the orchestration of radical interventions with PMN‐modulating strategies to counteract metastatic niche potentiation, thereby achieving a survival‐driven equilibrium between locoregional tumor control and systemic recurrence prevention. Concurrently, the development of early detection biomarkers and targeted therapeutic strategies (nanoparticle‐mediated drug delivery, specific signaling pathway inhibition, or natural product‐based PMN modulation) holds promise for shifting the paradigm from traditional “reactive metastasis management” to proactive “interception at the source.” The establishment of this multidimensional clinical framework will propel the prevention and treatment of lung metastases, toward a precision medicine era characterized by preemptive and individualized therapeutic approaches.

### 5.1 PMN Alterations Following Primary Tumor Therapy

#### 5.1.1 Impact of Surgical Treatment of the Primary Tumor on the Lung PMN

**FIGURE 2:**
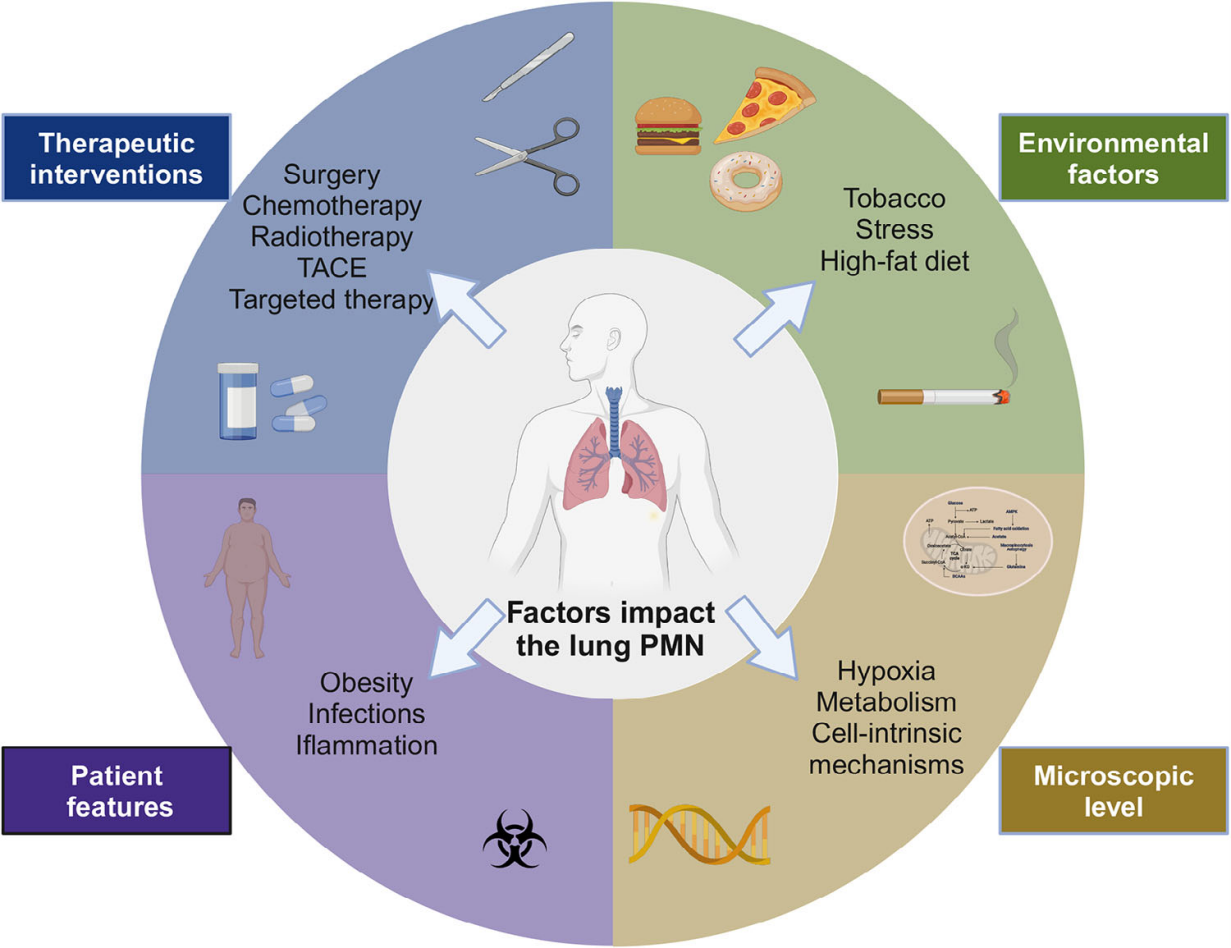
A multitude of cell‐intrinsic and systemic factors impact the patient and the lung PMN. This illustration portrays the intricate and multilayered factors in lung PMN. This includes tumor cell‐intrinsic and ‐extrinsic factors, such as underlying obesity, infection, and inflammation. Environmental factors are also emerging as important modulators of TME, including smoking and other carcinogens. Finally, therapeutic interventions, including surgery and chemo‐, radio‐, and targeted therapy, can impact the lung PMN. Therefore, rational combinations to effectively target this multitude of factors will be critical for developing effective, long‐term control of the disease.

Tumor surgery can induce an inflammatory trauma that exacerbates the colonization of residual tumor “seed” in secondary sites, thus facilitating postoperative metastasis within the PMNs “soil” [161]. Surgery‐induced ECM remodeling renders the lungs more susceptible to metastasis. Reducing the activity or levels of LOX after surgery decreases lung metastasis and enhances survival. This highlights the potential of LOX inhibition as a strategy to reduce the risk of metastasis following surgical interventions [89]. Hepatic ischemia–reperfusion injury represents the principal outcome of surgical stress during hepatectomy. Functionally, plasmacytoid DCs that produce IFNα stimulate the recruitment of CX3CR1+ MDSCs through hepatocyte IRF1/CX3CL1 signaling, resulting in tumor recurrence after hepatectomy in HCC. By targeting plasmacytoid DCs and the IFNα/CX3CL1/CX3CR1 pathway, it may be possible to inhibit HCC recurrence induced by surgical stress, thereby reducing postoperative immunosuppression [162]. In terms of function, plasmacytoid DCs that produce IFNα stimulate the recruitment of CX3CR1+ MDSCs through hepatocyte IRF1/CX3CL1 signaling, subsequently promoting tumor recurrence following hepatectomy in HCC. The choice of surgical approach, whether it is laparoscopic liver resection (LLR) or open liver resection (OLR), can impact the recurrence rate of HCC derived from lung metastasis. LLR, compared with OLR, is associated with significantly reduced levels of GM‐CSF, IL‐6, IL‐8, and MCP‐1. This decline in systemic inflammation after LLR may contribute to better short‐term and long‐term outcomes, making LLR a potentially important mechanism for improving overall outcomes compared with OLR [163]. The immune microenvironment undergoes changes following splenectomy, impacting not only primary tumors but also premetastatic and metastatic locations. Following splenectomy, there is a decrease in the accumulation of tumor‐infiltrating DCs, TAMs, and TANs in the metastatic microenvironment. Furthermore, the immune composition of the PMN in the lungs undergoes alterations, potentially leading to reduced metastases [164]. In the realm of solid tumor management, a multitude of treatment modalities exists, with each approach exhibiting distinct impacts on lung metastasis (Figure 2). Consequently, in the clinical setting, it becomes imperative to carefully evaluate the advantages and disadvantages of these methods, ensuring precision in the selection of appropriate treatment approaches.

#### 5.1.2 Impact of Systematic Treatment of the Primary Tumor on the Lung PMN

Furthermore, it is clear that conventional treatments, such as chemotherapy and radiotherapy, lead to alterations in the TME, influencing their therapeutic effectiveness in a cancer cell‐extrinsic manner, either enhancing or obstructing the response [165].

The role of the chemotherapy drug cisplatin in lung metastasis is indeed controversial. On one hand, there is evidence suggesting that chemotherapy drugs can facilitate the accumulation of miR‐29a‐3p in the exosomes released by lung tumor cells, which contributes to the inhibition of lung metastasis [27]. On the other hand, it has been observed that chemotherapy‐elicited EVs are enriched in ANXA6, which promotes the establishment of lung metastasis. Additionally, administration of chemotherapy can trigger neutropenia and lead to the formation of NETs, thereby promoting the development of lung metastasis. This suggests a potential negative effect of chemotherapy in promoting metastasis [34, 104, 166]. Overall, the role of chemotherapy drugs in lung metastasis is complex, and further research is needed to fully understand their impact on metastatic processes. Changes in TME induced by radiation therapy can also promote metastasis [167]. Irradiation of primary tumor sites resulted in a notable increase in MDSCs in the spleen, along with the accumulation of PMN components in the lungs, thereby selectively enhancing pulmonary metastasis [33]. Moreover, the application of transcatheter arterial chemoembolization in HCC patients significantly upregulated miR‐4508 in plasma exosomes, thereby promoting lung PMN formation [24].

### 5.2 Early Identification Against Lung PMN

**FIGURE 3:**
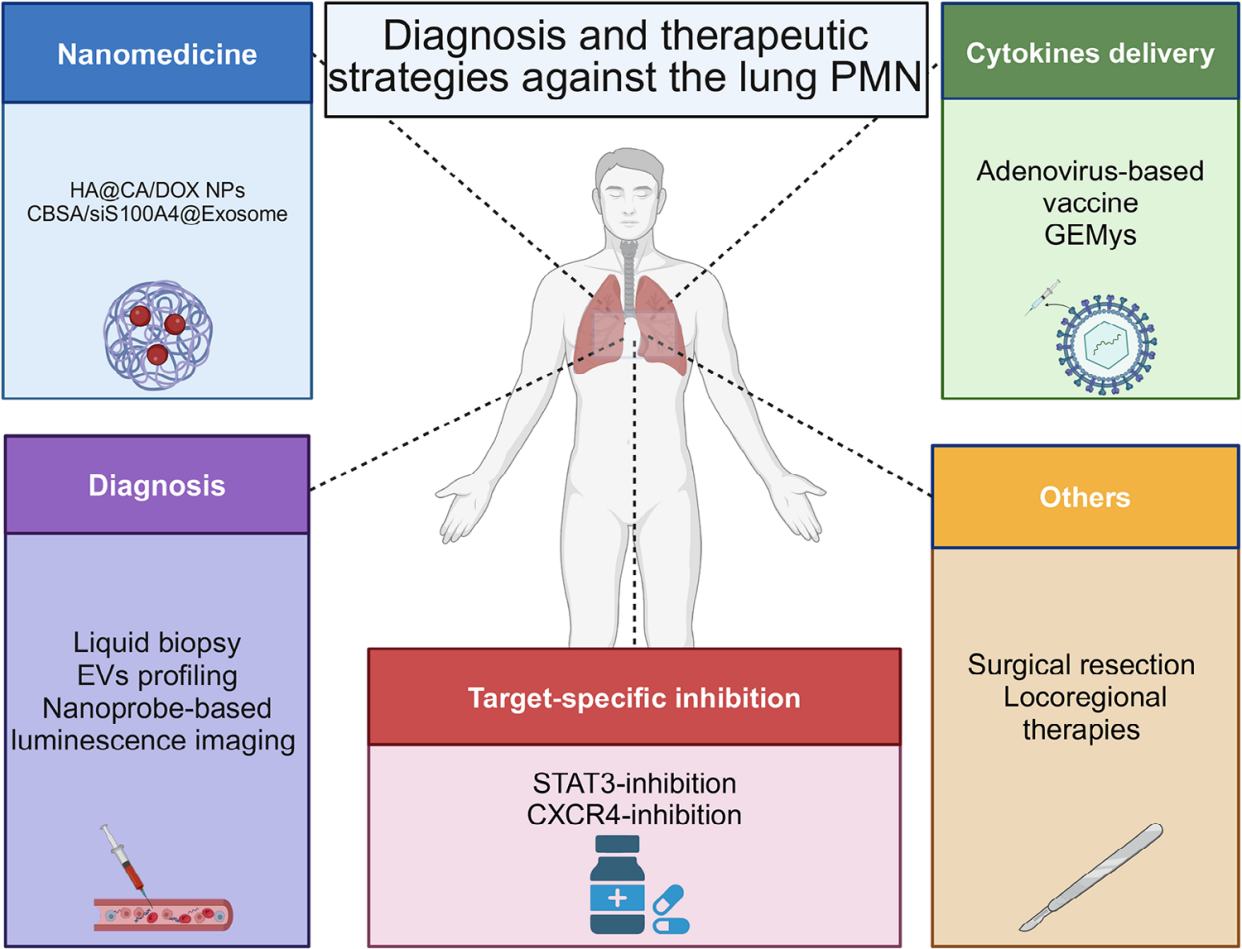
Diagnosis and therapeutic strategies against lung PMN. Advanced detection, such as liquid biopsy, helps identify the formation of lung PMN. Additionally, nano drug‐loading composite technology was used to target lung PMN. Several signaling pathway inhibitors have been developed and experimentally demonstrated to inhibit lung metastasis. Delivering cytokines is an effective strategy to prevent the formation of PMN in the lung. Finally, other novel therapeutic strategies have demonstrated promising efficacy in the lung metastasis. GEMys, genetically engineered myeloid cells.

Currently, most treatments for solid tumors do not effectively cure lung metastasis. Therefore, it is essential to investigate diagnostic methods that could identify metastasis in its early phases, when it may be more treatable (Figure 3). Due to its invasive nature and limited tissue sample, conventional tissue biopsy fails to capture tumor heterogeneity or track dynamic tumor progression. In contrast, liquid biopsy, being noninvasive, enables repeated analyses for real‐time monitoring of tumor recurrence, metastasis, and treatment responses. The advancement of new molecular techniques has yielded promising outcomes in the detection of CTCs and circulating tumor DNA [168, 169]. Moreover, in situations where obtaining tissue biopsies is unfeasible, EV profiling can be conducted to gain a deeper understanding of the patients' condition [170]. The lipid bilayer of EVs protects molecular markers such as proteins, lipids, and nucleic acids from both cancer and stromal cells, ensuring their stability [23, 171]. Using in vivo murine models with green fluorescent protein‐tagged tumor‐derived EVs (TEVs) and gene expression analysis of TEV‐capturing cells at distant metastatic sites, a detailed temporal and molecular analysis of TEV contributions to premetastatic and metastatic niches was developed [21]. A novel approach has been developed for the early detection of lung metastasis through the use of self‐illuminating nanoprobes to visualize pulmonary neutrophil infiltration via luminescence imaging [172, 173, 174]. Remarkably, the luminescence imaging strategy using nanoprobe targeting showed significantly superior performance compared with positron emission tomography/computedtomography (PET/CT) imaging modalities in the mouse model under investigation [128, 175]. The identification of MDSCs releasing S100A8 and S100A9 in the PMN led to the creation of S100A9‐specific antibody‐based single‐photon emission computed tomography (SPECT) for whole‐body imaging, enabling visualization of the premetastatic lungs [126]. Recent research and clinical trials comprehensively investigate the impact of environmental factors (e.g., smoking, NCT01182519) and lifestyle habits (e.g., circadian rhythm disruption, NCT05988970) on the lung PMN [176, 177, 178]. Furthermore, we have conducted multiple prospective trials focusing on early prediction of metastatic risk and dynamic monitoring through liquid biopsy and combined radiomics approaches (Table 3). Given the current limitations in diagnostic precision, we anticipate future advancements in biomarker discovery. Additionally, we are pursuing exploratory studies in targeted therapies.

**TABLE 3:** Clinical trials investigating the lung PMN.

NCT number	Conditions	Study type	Sponsor	Study start	Recruitment status	Study completion (actual/estimated)	Enrollment
NCT01182519	Breast cancer	Observational	Memorial Sloan Kettering Cancer Center	2010	Completed	2016	400
NCT06472804	Non‐small cell lung cancer	Observational	Mayo Clinic	2019	Recruiting	2026	60
NCT05988970	Lung cancer	Interventional	Institut Claudius Regaud	2024	Recruiting	2025	27
NCT03721120	Lung cancer	Interventional	Centre Leon Berard	2019	Completed	2023	319
NCT05187767	Lung cancer	Observational	University of Roma La Sapienza	2022	Unknown status	2025	80
NCT05846594	Lung cancer/gastrointestinal cancer	Interventional/phase 4	Hoffmann‐La Roche	2023	Completed	2024	466
NCT01722903	Colorectal cancer	Observational	Milton S. Hershey Medical Center	2012	Completed	2015	25
NCT05101655	Osteosarcoma	Observational	Ruijin Hospital	2020	Completed	2022	60
NCT03108677	Osteosarcoma	Observational	Ruijin Hospital	2017	Active, not recruiting	2023	90
NCT02507778	Lung cancer	Interventional	Rabin Medical Center	2015	Unknown status	2019	40
NCT00003901	Non‐small cell lung cancer	Interventional/phase 3	Alliance for Clinical Trials in Oncology	1999	Completed	2011	1310
NCT03681873	Metastatic malignant neoplasm in the lung	Interventional	Children's Hospital Medical Center, Cincinnati	2018	Completed	2021	78
NCT03242993	Non‐small cell lung cancer	Interventional/phase 1	University of Lausanne Hospitals	2017	Completed	2019	15
NCT03164486	Metastatic malignant neoplasm in the lung	Interventional/early phase 1	University of California	2016	Active, not recruiting	2024	27
NCT06358222	Non‐small cell lung cancer	Observational	Shanghai Chest Hospital	2023	Recruiting	2027	200
NCT05425134	Non‐small cell lung cancer	Observational	Shanghai Pulmonary Hospital	2022	Unknown status	2023	5000
NCT05834413	Lung cancer	Interventional	Shanghai University of Traditional Chinese Medicine	2023	Not yet recruiting	2026	367
NCT03198117	Non‐small cell lung cancer	Interventional	Qidong Gaitianli Medicines Co., Ltd	2017	Terminated	2017	6
NCT01176669	Breast cancer	Interventional	Fudan University	2010	Phase 2	2014	60

*Data sources*: www.clinicaltrials.gov.

### 5.3 Therapeutic Strategies Against Lung PMN

#### 5.3.1 Nano Drug‐Loading Composite Technology

Initially, due to the intricate composition of the PMN, hindering lung metastasis by focusing on a singular element may prove challenging. As a result, combining multiple key cellular mechanisms and molecular signaling pathways, such as those employed in combination therapy, could potentially be more effective in preventing metastasis.

Although facing numerous challenges, there is considerable potential in the increasing range of approaches for targeting the TME therapeutically, as highlighted in a recent review [179]. These include therapies such as nanomedicine, targeted therapy, cytokine delivery, and novel therapies.

Utilizing nano drug‐loading composite technology can bring about the practical implementation of therapeutic strategies in a clinical setting. Nanomedicines, with their hydrophilic and hydrophobic segments, promote the depletion of MDSCs by inducing their differentiation in the lungs and tumors, thereby modulating the inflammatory and immunosuppressive microenvironment and inhibiting PMN formation [180, 181]. Micelles further enhance these effects through MDSC modulation [182, 183]. When combined with anti‐inflammatory agents and chemotherapy, these drug delivery systems effectively reduce postoperative recurrence and pulmonary metastasis. Additionally, nano‐blankets and nanovesicles can prevent fibroblast activation, thereby reducing the creation of a prometastatic environment for circulating lung tumor cells [27, 182]. The biomimetic nanoparticles, specifically cationic bovine serum albumin (CBSA) conjugated with siS100A4 and exosome membrane‐coated nanoparticles (CBSA/siS100A4@Exosome), enhance drug delivery to the lung PMN. These nanoparticles demonstrate remarkable gene‐silencing effects, significantly inhibiting the growth of malignant breast cancer cells [184]. Surgical tumor resection often triggers intense inflammatory responses, allowing CTCs to implant in the PMN, leading to recurrence and pulmonary metastasis. An injectable hydrogel applied to the resection cavity effectively prevented both regional and metastatic recurrence [185]. This approach, which combines oncolytic immunotherapy and inflammation reduction, offers a promising solution for postoperative metastasis prevention.

#### 5.3.2 Target‐Specific Inhibition

Employing a combination treatment approach that induces immunogenic cell death while inhibiting CXCR4 aims to prime the TME and enhance the efficacy of anti‐PD‐L1 therapy. This method successfully triggered the T cell response within primary tumors by amplifying tumor immunogenicity to attract T cells, eliminating the physiological barriers related to intratumoral fibrosis and collagen for increased T cell infiltration, and decreasing immunosuppressive cells to rejuvenate T cells. Concurrently, this strategy effectively thwarted the development of PMN in distant lung tissues [166]. The targeted blockade of CXCR4 and inhibition of STAT3 exhibit promising clinical prospects as treatments for eradicating organ metastasis in advanced HCC [186].

#### 5.3.3 Deliver Cytokines

One approach to enhance the effectiveness of anticancer therapies is to strategically deliver cytokines [187]. Cytokines (e.g., IL‐12) that can recruit immune cells to the tumor or premetastatic sites continue to be tested in therapeutic oncology settings. Some studies present various strategies for administering IL‐12, each with its unique mechanisms and advantages. These techniques include the utilization of lipid nanoparticles, adenovirus‐based vaccine, intratumoral administration, and genetically engineered myeloid cells (GEMys) [188]. IL12‐GEMy treatment reverses immune suppression in the PMN by activating antigen presentation and T cell activation, resulting in reduced metastatic and primary tumor burden [189]. A fusion of the cytokine LIGHT with a vascular‐targeting peptide induces high endothelial venules and lymphocyte clusters, enhancing the sensitivity of refractory lung metastases to anti‐PD‐1 checkpoint inhibitors [190]. The effectiveness of various treatment modalities (such as surgical resection, nonsurgical locoregional, and systemic therapies) has been demonstrated in combating lung metastasis (Figure 3). Consequently, personalized therapeutic strategies are customarily selected by the individual requirements of each patient.

#### 5.3.4 Natural Product‐Based PMN Intervention

The “seed and soil” hypothesis of metastasis aligns conceptually with the traditional Chinese medicine (TCM) axiom, “where pathogenic factors accumulate, the vital Qi is inevitably deficient,” and PMN discovery empirically bridges both theories [191, 192]. As a holistic system rooted in “preventive treatment,” TCM offers unique perspectives on metastatic propensity through multicomponent, multitarget mechanisms—altering the gut microbiota composition, regulating immunosuppressive mediators (e.g., recruiting MDSCs, TAMs polarization), and attenuating systemic inflammatory priming [193, 194]. We underscore the need for rigorous integration of TCM with conventional therapies, guided by evidence‐based formulations and biomarker‐driven strategies, while explicitly addressing critical challenges, including pharmacokinetic validation, standardization of bioactive components, and biomarker‐anchored efficacy assessment within the framework of modern PMN research.

## 6 Conclusions and Perspective

Metastases to the lungs are frequently observed in solid tumors such as HCC, breast cancer, osteosarcoma, and melanoma, carry an unfavorable prognosis and are difficult to treat. Systematically, extrathoracic malignancies alter the pulmonary microenvironment to facilitate the establishment and growth of DTCs to generate secondary lung tumors. It is approximated that around 70% of cancer patients have died of tumor metastasis. Within this group, an estimated 20–30% endure the burdens of pulmonary metastasis [195]. Numerous cell types and factors derived from the host are recruited to the local lung stroma, contributing to a favorable secondary microenvironment for DTCs.

**FIGURE 4:**
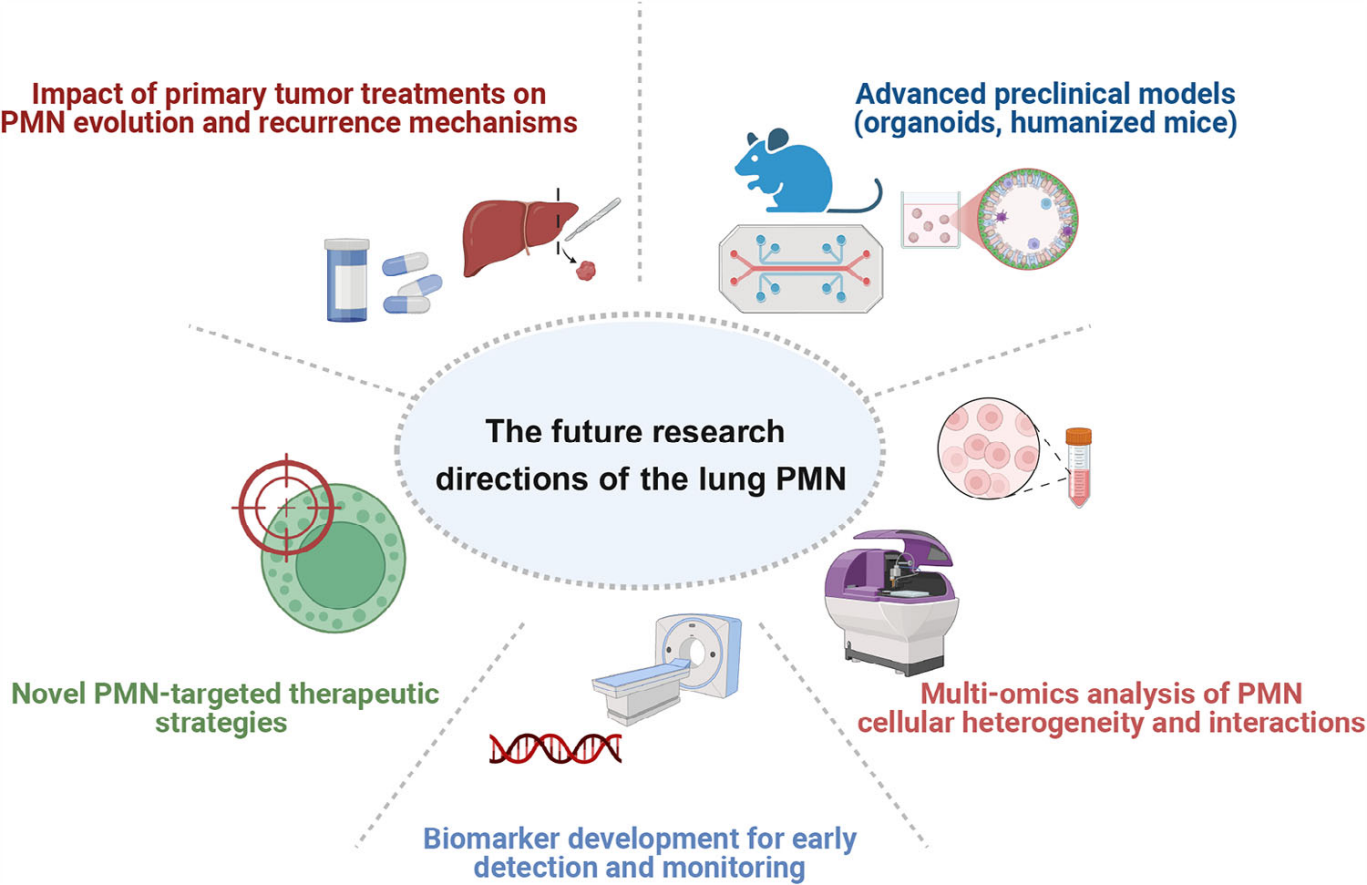
Evolving frontiers in lung PMN research: Conceptual frameworks and translational opportunities. Future research in lung PMN biology will prioritize advanced preclinical models (e.g., organoids, humanized mice) integrated with multiomics technologies to dissect cellular heterogeneity and interactions, while developing liquid biopsy‐based biomarkers for early metastatic risk stratification and dynamic surveillance. Concurrently, therapeutic strategies targeting PMN‐specific vulnerabilities and investigations into primary tumor treatment‐induced PMN remodeling are critical to unravel recurrence mechanisms and refine intervention paradigms.

Despite the advancements in clinical and preclinical research, some questions remain unanswered. For example, it is imperative to develop better lung metastasis models. Over several decades, investigations into the TME in lung metastasis have predominantly relied on in vitro 2D cell cultures as well as in vivo xenografts or genetically engineered animal models. However, conventional models have limitations. Recently, advances in bioengineering have led to platforms for large‐scale testing, including ex vivo organoids and tissue slices that closely mimic organ‐specific TME (Figure 4). The emergence and application of organoids and organ chip technology have enhanced the way for the enhanced utility of lung organoids, which emulate the human lung functionality with greater fidelity. This advancement holds immense promise for modeling pulmonary TME and facilitating drug screening and development [196]. The development of patient‐derived 3D lung organoids and organ chips technology has revolutionized the study of PMN formation. In the realm of research into brain metastasis of lung cancer, multiorgan microfluidic chips have emerged as a pragmatic alternative for unraveling the pathogenesis of this condition. Concurrently, the discovery of AKR1B10 has illuminated its role as a diagnostic biomarker and a prospective therapeutic target for NSCLC brain metastasis [197]. We expect that organ chips hold promise as models for examining the interaction between the TME and cancer cells during the lung metastasis process. Future efforts should integrate microfluidic “niche‐on‐chip” systems with AI‐driven predictive algorithms to map therapeutic vulnerabilities across lung PMN developmental stages.

In addition, identifying all cell types in PMN poses a significant challenge. ScRNA‐seq (GSE263963) and flow cytometry have identified distinct cellular states within the lung PMN cellular states during breast cancer metastasis, including mirrored CD14+ ‘‘activated’’ myeloid cells and elevated cytotoxic NK cell proportions. Longitudinal scRNA‐seq atlas revealed a TLR–NF‐κB inflammatory program enacted by both peripherally derived and tissue‐resident myeloid cells that correlated with PMN formation. Spatiotemporal modulation of the IGF1 and CCL6 signaling axes across diverse cellular compartments during metastatic evolution reveals immunotherapy‐targetable pathways for metastasis interception [198]. Future integration of chromatin accessibility (ATAC‐seq) and spatial metabolomics will decode the epigenetic drivers of lung PMN plasticity.

Finally, while PMN plays a crucial role in lung metastasis, the exact detection of PMN emergence and its scope during metastasis poses a challenging issue. As anticipated, the metastatic microenvironment displayed protumor characteristics. Contrary to conventional beliefs about metastasis, the premetastatic and initial metastatic environments consisted of immune cells exhibiting an antitumor phenotype. The shift from antitumor to protumor characteristics correlated directly with the actions of neutrophils and monocytes during these specific time points. The medium conditioned by PMN‐derived cells effectively impeded the proliferation and movement of tumor cells in vitro. Furthermore, depleting premetastatic neutrophils and monocytes in vivo worsened survival outcomes, thereby confirming the antitumor characteristics of the developing microenvironment [6]. Emerging evidence demonstrates the critical role of CT in detecting lung PMN alterations prior to radiologically visible lung metastases. Preclinical studies in 4T1 breast tumor‐bearing mice revealed increased lung density on micro‐CT correlating with inflammatory microenvironment remodeling, validated histologically. Clinically, radiomic analysis of 29 breast cancer patients identified 26 significantly altered features (e.g., run percentage, AUC = 0.85–0.95) in premetastatic regions versus contralateral tissues, with five features linearly correlated to tumor progression [199, 200]. These findings confirm CT's capacity to quantify PMN‐associated stromal changes, such as ECM remodeling and immune cell infiltration, before metastatic colonization. Delta‐radiomics models quantifying lung texture changes on serial CT scans further enable noninvasive PMN monitoring. Emerging multimodal approaches are revolutionizing PMN detection, combining advanced imaging with liquid biopsy to intercept metastasis at its earliest stages (NCT05846594). Next‐generation platforms must standardize EV isolation protocols and validate multianalyte signatures in international cohorts. The convergence of advanced diagnostics, including S100A8/A9‐SPECT imaging (4T1.2 vs. control: 0.95 vs. 0.45 %ID, *p* < 0.001), redefines oncology from reactive treatment to proactive niche interception. This modality also quantifies therapeutic efficacy, as shown by reduced PMN activity post‐CCL2 blockade (2.0 → 1.4 %ID). Building on these findings, we propose that key priorities include standardizing cross‐platform biomarker quantification and validating these tools in adaptive trials to guide niche‐directed therapies like CCL2/PD‐1 inhibitors [126]. This evolving paradigm transforms imaging biomarkers from descriptive tools into therapeutic compasses that anchor metastatic interception in mechanistic precision. By bridging the nanoscale molecular sensitivity of SPECT with the radiomic spatial resolution of CT to detect subclinical PMN before radiographic metastasis, thereby transforming interception into a precision oncology reality.

In the future, significant progress is anticipated in harnessing the potential for targeting lung PMN in the years ahead. Additionally, we highlight advancements in live imaging, comprehensive profiling at multiple scales, and analysis of bulk gene expression data to map complex microenvironments. Converging preclinical models, multiomics technologies, and clinical insights have positioned PMN interception as a transformative oncology frontier. The key priorities include validating AI‐guided therapeutic combinations in adaptive platform trials and establishing PMN‐centric endpoints for drug approval. By decoding the niche spatiotemporal rules, this paradigm shift offers potential to transform metastatic cancer into a preventable disease.

## Author Contributions

Chenghao Cao, Di Lu, Huigang Li, Shen Pan, Jianyong Zhuo, Peiru Zhang, Chiyu He, and Zuyuan Lin designed and prepared the manuscript. Chenghao Cao, Di Lu, and Xiao Xu drew and revised the figures. Xiao Xu, Xuyong Wei, Zhe Yang, Songmin Ying, and Shusen Zheng revised and supervised the manuscript preparation. All authors read and approved the final manuscript.

## Conflicts of Interest

The authors declare no conflicts of interest.

## Ethics Statement

The authors have nothing to report.

## Data Availability

The authors have nothing to report.
